# Working Silicate Cement

**Published:** 1915-08

**Authors:** Charles E. Jones


### WORKING SILICATE CEMENT.

BY CHARLES E. JONES, D.D.S.

While the term "silicate cement" seems to encompass our understanding of the relative characteristics of cements of the type under discussion, it would seem quite in place to here specify by what term some men, whose knowledge of the chemical arrangement of the elements contained in such products, believe a compound of this type should be called.

The tendency of the manufacturer to disguise his product with such misleading terms as "synthetic porcelain" and other ambiguous names has led because of our rather comprehensive interpretation of the meaning of the term porcelain, to an incorrect understanding of the composition of the materials in these cements. We should decry the use of names that misrepresent the character of these products.

The dental chemist has had in the preparation of the ingredients of a silicious cement one of the most difficult of tasks. He has been called upon to produce a substance dissimilar in character to any other product in the cement line. He has been circumscribed to a very limited field, from which to draw the elements known to possess qualities necessary to fulfill the severe requirements demanded by the profession. And while I do not consider his work completed, I can not refrain from here emphasizing the importance of his efforts in this particular field.

I do not think that any producer of cements of the type under discussion has made the claim that silicious cements are permanent in the light of our understanding of permanency as represented by gold as a filling material. Nor do I believe that the honest experience of any user of these cements would, up to this time, justify an assertion encompassing the significance a remark of this character would entail. In view of the fact that we are not dealing with a permanent material, as it is now used, and being of the belief that a much more permanent and durable silicious body may be prepared, by paying strict attention to our technic in handling these ingredients the following, somewhat impromptu, discussion of the subject is offered.

If we can increase the durable and lasting properties of these cements by manipulative procedures, it is obvious that we should adopt every known means to bring about this end. Thus we will offset many of the disappointing results which we would otherwise experience.

If proper preservation of the ingredients; care in selecting shades; thorough and sufficient mixing; correct cavity preparation; proper insertion and finishing, along with subsequent protection from moisture coupled with care and judgment used in selecting the place for its use are things which will increase the permanency of these materials, it is quite apparent that they should be the important considerations included in our attempt at the successful handling of these restoring agents.

I take it that all of you have used silicious cements in rather an empirical way. This inference has been caused by the meagre amount of information we understand nearly all of the members of the profession have relative to the ingredients, formulas, etc., of these products. And while the problem before the manufacturer is a chemical one, it does not necessarily follow that the manipulation of the powdered silicates and the accompanying liquids means a continuation of extensive chemical processes. That part remaining for the dentist to perform in the manipulation of cements is devoid of much chemical reaction. For example: The liquid of a cement is a solution of phosphoric acid loaded to almost saturation with modifiers. In fact there is such a small quantity of the phosphoric acid left free and uncombined that there are but few hydrogen (H) elements to displace when brought in contact with the basic elements of the powder and very little chemical reaction remains for the dentist to induce in the preparation of his mixes.

I am of the belief that the problem before the dentist, in the proper handling of cements, involves a thorough understanding of their physical characteristics or the chemical arrangement of their ingredients. It would seem, from my viewpoint, that if the user possessed enough information to carry into effect the instructions of a reliable manufacturer, together with the proper attention to detail, that he would be sufficiently well prepared to use successfully these newer forms of filling materials in so far as success may be attained with them.

There seems to be a degree of satisfaction manifested by us as displayed by our contentions about things of which we are ignorant. But I always feel that if a man knows literally just what he is doing or trying to do, that he will come nearer completing the task than if he occasionally staggers blindly upon an accomplishment without really knowing just how he achieved the thing desired. I, therefore, believe that the elemental factors concerned in cement mixing should be understood, and with that end in view, I shall try to make clear just what I think transpires when a silicate powder and a cement liquid are brought together.

While I have a keen desire to deal with the subject of silicious cements from the standpoint of their physical properties alone, I do not believe that I shall impose discomfort upon you when it shall be found necessary to refer to chemical activities by way of substantiating contentions in the discussion, or to assist in elucidating points germane to the subject. I take it that the members of the profession are quite familiar with the various makes of silicious cements now offered by the several manufacturers under the various names, and when it shall become necessary, in this discussion, to make special reference to the product of any individual manufacturer, I am sure that you will at once understand that the application used signifies, in such reference, a general class represented by a large family grouping known in their cemented or mixed forms either as *silicious or calcium phosphate* cements, in which the liquid is an aqueous solution of phosphoric acid and its modifiers, in the form of phosphates, and that, the portion known as the powder is a silicate substance containing Aluminum Oxid, Calcium Oxid, Silicon Dioxid, Phosphoric Pentoxid or a similar flux, in combination with such elements as Lithium, Berilium, etc., modified with rare earths and pigmented with metallic oxids.

It may seem, I know, quite presumptuous on my part to attempt to present views other than those held by the manufacturers of these cement ingredients, but I am compelled to do so in view of the results I have observed from rather a long line of laboratory experiments and clinical observations.

In the manufacture of these silicate substances, which subsequently become the powders of the cements, a very refractive substance of almost unknown chemical relations is produced, but the manufacturer has for his purpose the preparation of a material of practically insoluble properties. As a material is produced upon which an acid of the comparative avidity of phosphoric acid has no action, it becomes necessary to add an agent which will permit of a reaction between an acid of this type and the silicate mass. Calcium is the agent used, and it therefore becomes the controlling factor.

The ingredients of a silicious cement, as has been before stated, consists in their unmixed state and as received from the manufacturer, of finely ground silicates and aluminates, and a liquid representing an aqueous solution of ortho-phosphoric acid and modifiers, in the form of aluminum, zinc or perhaps other phosphates. We might sum up our present understanding of a silicate powder by saying that it is a fused mass of basic oxids, with silica and aluminum as the chief ingredients, and upon which is depended along with the phosphorous, for translucency. The calcium content is incorporated in excess, chemically, so as to permit of the ready action of an acid like phosphoric upon it to form a crystallizing, or agglomerating mass with subsequent basic hydration of the silicates by the water formed from the reaction of the acid upon the basic oxid.

Orthophosphoric acid, being tribasic, dissociates to form three kinds of salts. We would, therefore, expect to find calcium salts of either the primary, $CaH_4(Po_4)_2$ or the secondary, $2CaHPo_4$; or the tertiary or normal variety $Ca_3(Po_4)_2$ in silicious cement mixes. Ostwald and others inform us that phosphoric acid dissociated into the radicals H and $H_2Po_4$ more frequently than in any other forms, we therefore would expect the radical dihydrogen phosphate to combine with a divalent metal or base to form a primary phosphate $CaH_4(Po_4)_2$ (monocalcium tetrahydrogen diphosphate) a salt known to be very soluble. The soluble part of a cement, or that part representing a phosphate salt—or the agglomerating mass or binder—is regulated by the extent of the action of the basic oxid of calcium upon the trivalent phosphoric acid ($H_3Po_4$). To explain; if in bringing the modified aqueous solution of phosphoric acid ($H_3Po_4$) in juxtaposition with the calcium oxid, you permit and promote by spatulation of the entire displacement of the hydrogen radicals of the acid you have formed a normal or tertiary $Ca_3$ $(Po_4)_2$ which is an insoluble salt and the cement mass will not disintegrate, but on the other hand where you bring into contact, without spatulation, the acid liquid and the powder a primary or most frequently a secondary salt is formed empirically represented as $CaHPo_4$ which is quite soluble and which increases the instability of the cement or agglomerating portion of the mix.

While a combination of calcium and the group $Po_4$ in any one of the three possible salt forms may be quite insoluble, it is well known that the normal salt $Ca_3(Po_4)_2$ is least soluble of any. It, therefore, seems quite obvious that whenever possible this tertiary or normal salt should be formed, as has been previously stated in this discussion. Orthophosphoric acid, ($H_3Po_4$) dissociates invariably into the ions H and $H_2Po_4$. The divalent calcium element would, if brought into contact with but two molecules of the phosphoric acid, form $Ca(H_2Po_4)_2$ a primary or more soluble salt than the normal calcium phosphate.

It thus will be seen that the agglomerating mass, or that portion of the cement mix binding the admixture together, is most always composed of a soluble salt; that is, where no effort has been made to induce further dissociation than the primary stage of salt formation.

Since it is our desire to increase the permanency of these silicious mixes, when they are used as restorations of tooth structure it might be well for us to here consider by what means this permanency may be brought about.

Normal calcium phosphate, as has been stated, is practically insoluble in water. It is obvious therefore, that the normal salt is the most desirable. Let us then look for some method or way of transforming this soluble salt, primary calcium phosphate, into the more insoluble form, normal calcium phosphate. *It is known that insoluble calcium phosphate may be formed when it is admixed with a soluble calcium phosphate in solution.* If this known reaction can be depended upon, that is, with the procedures we expect to put in operation in the mixing of our cements, I am of the belief that we can nearly always make an insoluble agglomerating mass out of the product first produced when the phosphoric acid and basic calcium oxid is brought together by thorough and prolonged *SPATULATION*. The process of spatulation, acting as a mechanical means of bringing those portions, or combinations of the divalent calcium and dihydrogen phosphate into a sufficiently close physical relationship to permit and promote of further dissociation of the hydrogen atoms still remaining attached, or withheld in the primary or even secondary salt formations.

As it is our desire to have this normal phosphate formed, because of its insolubility and realizing that the heavy syrupy liquids, because of the low water content (in proportion to its dissolved ingredients) possesses very little dissociating power, and thus fail to act as a suitable vehicle for carrying the basic calcium oxid in contact with the acid, I am led to suggest making the mixes upon a slab approximating sixty-five degrees of temperature, bringing the powdered silicates in small proportions into contact with the liquid with the aim of producing a normal calcium salt, by dissociating the hydrogen ions of two molecules of phosphoric acid with three molecules of calcium; depending upon spatulation to dissociate the radicals instead of permitting a slower and not so complete reaction, which invariably results in the formation of an acid salt. The mechanical procedure of spatulation, we thus would expect to serve as would a liquid having high dissociating properties, and of increasing the encounters between the molecules of the reacting agents.

In order to impress you with the necessity of doing this operation of spatulation as suggested, I wish to here state that polybasic acids of the variety used in cement making are dissociated slowly and that each successive displacement of the combining weight of the hydrogen is done so with increasing difficulty. To illustrate this point, the first hydrogen displaced in the reaction between the tribasic phosphoric acid and the basic calcium ingredient of the silicate powder is produced with comparative ease and rapidity—calcium dihydrogen phosphate being the product—but when further dissociation is attempted a more difficult task is confronted, owing to the tendency of the divalent combination $H_2Po_4$ to remain closely united and difficult to dissociate. This phenomenon will help to explain why in hastily made mixes there is invariably formed, a primary or soluble salt of calcium, but that further dissociation can not be doubted. One or even both of the molecules of the hydrogen remaining in the monocalcium dihydrogen phosphate may be displaced, when this product in solution, and a secondary or tertiary salt of calcium formed by further spatulation or agitation, thus bringing the base calcium in contact with the divalent hydrogen phosphate ion while the liquid of the mix is still able to act as a vehicle in carrying the elements into sufficiently close chemical relation to permit of an interchange of their radicals. The truth of this contention has been satisfactorily demonstrated by testing the character of the salts formed in various ways of spatulation and further in noticing the acid reaction in cements that had been mixed and permitted to set for a long time.

For the sake of describing the process by which an insoluble salt may be formed in our cement mixes and in hopes that you may have a better understanding of just what we mean when we suggest prolonged spatulation in the early part of the cement mixing, the following is offered: I believe it is customary for us to introduce the correct amount of powder into a mix of a two drop size in at least five or six divisions until we have induced a putty like consistency advised by the manufacturers of cements of this character. The line of procedure about to be suggested is no different from that already well-known to you except that comparatively small portions of the powder are drawn into the liquid during the early stages of the mixing process. If you are in the habit of drawing a considerable quantity of the powder into the liquid in the first one or two portions utilized in the mix, I would advise you to divide these portions, for a two drop mix, into at least six portions. The powder being light and fluffy is readily absorbed by the liquid, thus during the process of spatulation the six or more small divisions of the powder are readily taken up and the mix remains in a semi-liquid state. We continue to add small additions of the powder with considerable spatulation until the powder begins to assume a creamy consistency and that extreme glaze or gloss that is noticed over the surface of the mix is practically lost, owing to a neutralization of the phosphoric acid. This difference, in mixing, between the plan now generally followed and the one suggested only includes that portion of the process indulged in early in the mixing period. The portions of the silicate powder added late in the mix, in order to obtain physical consistency, are made in the same manner as now practiced. It is only during the early admixing of the cement ingredients, and before one or more combining weights of the hydrogen of the acid has been dissociated, that we can hope to induce the chemical change, through which an insoluble agglomerating mass may be formed.

It is true that the prolonged spatulation will retard the setting properties of the finished mix, first, by inducing a loss of heat, formed through chemical reaction, by radiation into the mixing slab, and second, by disturbance of the process of crystallization manifested during the initial or early period of the chemical combining of the elements of the cement. But tests have proved that the retardation induced in mechanically disturbing the reacting agents is of no appreciable length of time. It will be observed that the prolonged spatulation, providing the reaction leads up to the formation of a normal calcium phosphate, will induce complete neutralization of the acid content of the liquids—thus obviating irritations due to still active portions of the acid, likely to be contained in the mix after its insertion into the tooth cavity.

While the silicious mix may set slower owing to the loss of heat during spatulation and prevention of any gross chemical reaction after the material has been inserted, I do not believe, nor does any test I have been able to use in my experimenting, cause me to believe that the filling becomes more susceptible to the disturbing action of the extraneous water contained in saliva, and which may come in contact with the unset mass. On the contrary, I am led to believe that the silicious mass is less subject to the deleterious action of the water content of saliva than when hastily mixed and inserted while chemical changes are still active between the acid and basic oxids. This might be explained by stating that by thorough spatulation a sufficient amount of water is formed as a product of the reaction of the acid upon the base to permit of a proper balance in the water of constitution in the crystallizing mass, with subsequent slow hydration of the silicates as explained in another portion of this discussion.

Believing then that it is possible to most always produce a normal calcium phosphate by thoroughly spatulating those small portions first brought into mix, it is to this one feature of our procedure in the preparation of these cements, that I wish to particularly call your attention.

The mixing slab should be thick, etched, glass absolutely clean, and free from deep scratches. The spatula should best be agate, and one with a sufficiently long working blade to reach nearly across the slab; it should also be thin enough to permit of transmitting a sense of feel at its working portion. The number of instruments for inserting the material should be ample and of suitable shapes and sizes; they may be of bone, agate, ivory, tantalum or any similar material. Steel should never be used for reasons you well know. If Berylite, or a similar silicate, that is known to have an energetic liquid or powder, or both, is to be used it will be well to cool the slab to as low a degree as atmospheric conditions will permit. The cold mixing slab becomes a necessity in those cements that heat quickly following chemical reaction between liquid and powder. The reduction of temperature may be appreciated when you are reminded of the fact that a raise of ten degrees F. in the temperature of the slab and cement ingredients thereon situated will increase the avidity of the acid one hundred per cent. It is also well known that a retarded chemical reaction in the mix will permit of greater incorporation of the cement powder. This may work a decided advantage in those cases where translucency is not desired, since a greater portion of the insoluble ingredients may be brought into the mix.

We are assuming that previous to making the mix you have arranged your instruments and accessories, such as matrices, etc. The colored variety of matrix serves best. Lavolin, vaseline, or any unctuous or oily substance for the purpose of lubricating and preventing adhesion of the cement to the surfaces of the matrix. Personally I like to use liquid lavolin. The matrix should be so attached that it will represent a rigid wall, against which to work and confine the plastic material. Pulps should be protected in deep seated cavities, by placing non-conductive agents between pulp and the silicious filling material. Pulps occasionally die under silicates, not so much due to any irritating action of the cement but due I think to encroachment upon pulp chambers without inducing an exposure as we would likely do in cavity preparations for gold.

The mix should be stiff enough, except in special instances, to permit forcible packing. The angles and undercuts should be first filled, followed by the insertion of a sufficient quantity of the cement to force that portion first placed laterally against the walls of the cavity and into definite position.

When the cavity has been filled to excess the matrix may be brought into final position and the filling compressed and retained until a partial set, at least, has been secured. At no time should the pressure upon the matrix be relieved until the filling material is no longer likely to move.

One important thing in connection with the use of the matrix is to always keep the filling material and matrix undisturbed until a firm set has been permitted to take place. This means that the matrix should be well secured in position before beginning the insertion of cement. [

am quite sure that insecure and early removals of the matrix cause more failures than any other feature of our faulty manipulation of this material.

After waiting at least ten minutes for the cement to partially crystallize the dam may be removed and the filling finished, with due regard to a desire to place all surfaces in a polished condition. This may be done with fine cuttle fish discs, or strips that have had incorporated in the grit, paraffin or some similar lubricant.

Wear by attrition seems to be the most frequent change occurring in cements of the variety under discussion, and since there is a possibility of minimizing the amount of loss in this respect, it might be well for us to here state that the greater the polish, the less surface exposed to attritional forces and the more lasting and durable will be the finished product. A rough or irregular filling presents immeasurably more surface to frictional and dissolving action than does the filling with smooth surfaces. The celluloid matrix seems to put an ideal finish on these fillings. On the occlusal surfaces a very thin piece of celluloid may be held in position and the filling burnished under it, spreading always with a burnishing-like motion toward the cavity margins. I have made sufficient experiments to at once determine in my mind that a filling which is kept well polished will outlast the one unpolished. This thing I have observed in fillings in the same mouth. It has been my custom to occasionally repolish the fillings when opportunity afforded. I have thus rendered a much more lasting service with these cements.

I know of nothing that the operator can do in working these cements that will so increase their durability as a high polish. High polish in this respect, means a series of fine scratches running in the same direction.—*Dental Review.*

